# Service Quality and Customer Satisfaction in the Post Pandemic World: A Study of Saudi Auto Care Industry

**DOI:** 10.3389/fpsyg.2022.842141

**Published:** 2022-03-11

**Authors:** Sotirios Zygiaris, Zahid Hameed, Mubarak Ayidh Alsubaie, Shafiq Ur Rehman

**Affiliations:** ^1^College of Business Administration, Prince Mohammad Bin Fahd University, Khobar, Saudi Arabia; ^2^Department of Management Sciences, University of Baluchistan, Quetta, Pakistan

**Keywords:** auto care, customer satisfaction, service quality, Saudi Arabia, pandemic (COVID-19)

## Abstract

The aim of this research is to examine the impact of service quality on customer satisfaction in the post pandemic world in auto care industry. The car care vendor in the study made effective use of social media to provide responsive updates to the customers in the post pandemic world; such use of social media provides bases for service quality and customer satisfaction. The study examined the relationship between service quality and customer satisfaction using the SERVQUAL framework. According to the findings, empathy, reliability, assurance, responsiveness, and tangibles have a significant positive relationship with customer satisfaction. Our findings suggest that it is critical for workshops to recognize the service quality factors that contribute to customer satisfaction. Findings also suggest that empathy, assurance, reliability, responsiveness, and tangibles contribute to customer satisfaction. Auto repair industry must regularly provide personal attention, greet customers in a friendly manner, deliver cars after services, notify customers when additional repairs are required, and take the time to clarify problems to customers. Furthermore, workshops must screen and hire courteous staff who can clearly communicate the services required to customers both in-person and online and effectively communicate the risks associated with repairs. Service quality seems to be aided by prompt services.

## Introduction

The previous studies on the effect of pandemic have focused on the behavior related to preventative measures to protect the health of the customers; however, less attention has been paid to the influence of pandemic on customer outcomes. To fill this gap, the SERVQUAL framework was employed to examine the changes in customers’ social media behaviors that have occurred since the pandemic was declared (Mason et al., 2021). In the post pandemic world, the parameters for customer satisfaction have changed considerably (Monmousseau et al., 2020; Srivastava and Kumar, 2021; Wu et al., 2021). Pandemic has made personal interaction more challenging (Brown, 2020). To be less vulnerable to becoming severely ill with the virus, customers prefer touchless digital mediums of communications. For example, Mason et al. (2021) concluded that pandemic has altered customers’ needs, shopping and purchasing behaviors, and post purchase satisfaction levels. Keeping in view the public healthcare concerns, the governmental pandemic mitigation policies also promotes touchless mediums for shopping; therefore, the role of social media as a communication tool stands to increase at a time when social distancing is a common practice; social media provides avenues for buyers to interact with sellers without physical contact. Thus, the use of social media gains critical importance, especially after the pandemic (Mason et al., 2021), and the businesses may find new opportunities to gain competitive advantage through their use of effective social media strategies.

The car care industry uses traditional means of customer communications. The company in this study made use of social media in improving their service quality through effective and safe communication with their customers. The use of social media to provide updates to customers played a significant role in improving service quality and satisfaction (Ramanathan et al., 2017). The company in the study used Snapchat to provide updates on the work, thus minimizing the customers’ need to physically visit the car care facility. This use of social media gave a significant boost to the responsiveness aspect of the service quality.

Service quality and customer satisfaction are important aspects of business since a company’s growth is largely dependent on how well it maintains its customers through service and how well they keep their customers satisfied (Edward and Sahadev, 2011). According to Chang et al. (2017); customer satisfaction is expected to result from good service efficiency, which will improve customer engagement and interrelationship. González et al. (2007) asserted that customer satisfaction is linked to high service quality, which makes businesses more competitive in the marketplace. This study uses the SERVQUAL framework to define service quality. This framework uses five dimensions to account for service quality, namely, tangibles, reliability, responsiveness, assurance, and empathy. Identifying issues in service and customer satisfaction can lead to high service quality. Furthermore, service quality can be characterized by analyzing the variations between planned and perceived service. Service quality and customer satisfaction have a positive relationship.

Recognizing and meeting customer expectations through high levels of service quality help distinguish the company’s services from those of its rivals (Dominic et al., 2010). Social media plays a critical role in shaping these service quality-related variables. Specifically, in the context coronavirus disease 2019 (COVID-19), where customers hesitated to visit auto workshops physically, the importance of online platforms such as auto workshops’ social media pages on Instagram and Facebook has increased, where customers try to get information and book appointment. For example, responsiveness is not only physical responsiveness but also digital means of communication. The car care company in this study uses social media as mode of communication with their customers due to physical interaction restriction caused by the pandemic.

Service quality becomes a critical element of success in car care industry because customer contact is one of the most important business processes (Lambert, 2010). Saudi Arabia is one of the Middle East’s largest new vehicle sales and auto part markets. Saudi Arabia’s car repair industry has grown to be a significant market for automakers from all over the world. As a result, the aim of this research was to see how service quality affects customer satisfaction in the Saudi auto repair industry.

This aim of this research was to answer the following research questions:

(i) What is the contribution of individual dimensions of SERVQUAL on customer perceived service quality of car care industry in Saudi Arabia?
(ii) What is the impact of perceived service quality on customer satisfaction in car care industry in Saudi Arabia?

## Literature Review

The concept of service has been defined since the 1980s by Churchill and Surprenant (1982) together with Asubonteng et al. (1996), who popularized the customer satisfaction theory through measuring the firm’s actual service delivery in conformity with the expectations of customers, as defined by the attainment of perceived quality, and that is meeting the customers’ wants and needs beyond their aspirations. With this premise, Armstrong et al. (1997) later expanded the concept of service into the five dimensions of service quality that comprised tangibles, reliability, responsiveness, assurance, and empathy.

Extant literature on service delivery focuses on the traditional emphasis on the contact between the customer and service provider (Mechinda and Patterson, 2011; Han et al., 2021). Doucet (2004) explained that the quality in these traditional settings depends on the design of the location and the behavior of the service provider. More recently, the proliferation of the internet has led to the emergence of the online service centers. In these cases, communication both in-person and online plays a critical role in the quality of service rendered. It follows that service quality in hybrid settings depends on quality of communications on social media as well as the behavioral interactions between the customer and the service provider (Doucet, 2004; Palese and Usai, 2018). These factors require subjective assessments by the concerned parties, which means that different persons will have varied assessments of the quality of service received.

### SERVQUAL Dimensions

Service quality has been described with the help of five quality dimensions, namely, tangibles, reliability, responsiveness, assurance, and empathy. Definitions relating to these variables have been modified by different authors. The relationship between various dimensions of service quality differs based on particular services.

### Tangibles

The tangible aspects of a service have a significant influence on perception of service quality. These comprise the external aspects of a service that influence external customer satisfaction. The key aspects of tangibility include price, ranking relative to competitors, marketing communication and actualization, and word-of-mouth effects (Ismagilova et al., 2019), which enhance the perception of service quality of customers (Santos, 2002). These aspects extend beyond SERVQUAL’s definition of quality within the car care industry settings. Thus, we proposed the following hypothesis:

**Hypotheses 1a:** Tangibles are positively related with perceived service quality.

### Reliability

Reliability is attributed to accountability and quality. There are a bunch of precursors that likewise aid basic methodology for shaping clients’ perspectives toward administration quality and reliability in the car care industry in Saudi (Korda and Snoj, 2010; Omar et al., 2015). A portion of these predecessors is identified with car repair benefits and includes the convenient accessibility of assets, specialist’s expertise level and productive issue determination, correspondence quality, client care quality, an exhibition of information, client esteem, proficiency of staff, representatives’ capacity to tune in to client inquiries and respond emphatically to their necessities and protests, security, workers’ dependability, more limited holding up time and quickness, actual prompts, cost of administration, accessibility of issue recuperation frameworks, responsibility, guarantees, for example, mistake-free administrations, generally association’s picture and workers’ politeness, and responsiveness. Despite the innovative changes happening in the car care industry and the instructive degree of car administrations suppliers in Saudi Arabia, car care suppliers in the territory are taught about the need to continually refresh their insight into the advancements in the area of vehicle workshops and the components of administration. Thus, we argued that reliability is important to enhance the perception of service quality of customers.

**Hypotheses 1b:** Reliability is positively linked with perceived service quality.

### Responsiveness

Responsiveness refers to the institution’s ability to provide fast and good quality service in the period. It requires minimizing the waiting duration for all interactions between the customer and the service provider (Nambisan et al., 2016). Nambisan et al. (2016) explained that responsiveness is crucial for enhancing the customers’ perception of service quality. Rather, the institution should provide a fast and professional response as to the failure and recommend alternative actions to address the customer’s needs (Lee et al., 2000). In this light, Nambisan summarizes responsiveness to mean four key actions, i.e., giving individual attention to customers, providing prompt service, active willingness to help guests, and employee availability when required. These aspects help companies to enhance the customers’ perception of service quality. Therefore, we proposed the following hypothesis:

**Hypotheses 1c:** Responsiveness is positively linked with perceived service quality.

### Assurance

Assurance refers to the skills and competencies used in delivering services to the customers. Wu et al. (2015) explains that employee skills and competencies help to inspire trust and confidence in the customer, which in turn stirs feelings of safety and comfort in the process of service delivery. Customers are more likely to make return visits if they feel confident of the employees’ ability to discharge their tasks. Elmadağ et al. (2008) lists the factors that inspire empathy as competence, politeness, positive attitude, and effective communication as the most important factors in assuring customers. Besides, other factors include operational security of the premises as well as the proven quality of the service provided to the customers. Thus, the assurance has significant contribution in the perception of service quality.

**Hypotheses 1d:** Assurance is positively related with perceived service quality.

### Empathy

Empathy refers to the quality of individualized attention given to the customers. The service providers go an extra mile to make the customer feel special and valued during the interaction (Bahadur et al., 2018). Murray et al. (2019) explains that empathy requires visualizing the needs of the customer by assuming their position. Murray et al. (2019) lists the qualities that foster empathy as including courtesy and friendliness of staff, understanding the specific needs of the client, giving the client special attention, and taking time to explain the practices and procedure to be undertaken in the service delivery process. Therefore, we proposed the following hypothesis:

**Hypotheses 1e:** Empathy is positively related with perceived service quality.

### Perceived Service Quality and Customer Satisfaction

Customer satisfaction refers to the level of fulfillment expressed by the customer after the service delivery process. This is a subjective assessment of the service based on the five dimensions of service quality. Customer satisfaction is important due to its direct impact on customer retention (Hansemark and Albinsson, 2004; Cao et al., 2018; Zhou et al., 2019), level of spending (Fornell et al., 2010), and long-term competitiveness of the organization (Suchánek and Králová, 2019). Susskind et al. (2003) describes that service quality has a direct impact on customer satisfaction. For this reason, this research considers that five dimensions of service quality are the important antecedents of customer satisfaction.

Service quality refers to the ability of the service to address the needs of the customers (Atef, 2011). Customers have their own perception of quality before interacting with the organization. The expectancy-confirmation paradigm holds that customers compare their perception with the actual experience to determine their level of satisfaction from the interaction (Teas, 1993). These assessments are based on the five independent factors that influence quality. Consequently, this research considers service quality as an independent variable.

This study attempts to quantify perceived service quality though SERVQUAL dimensions. We proposed that customers place a high premium on service quality as a critical determinant of satisfaction. Moreover, it is argued that satisfaction prompts joy and reliability among customers in Saudi Arabia. These discoveries infer that the perception of service quality is significantly related to satisfaction, and quality insight can be applied across different cultures with negligible contrasts in the result. Car care industry in Saudi Arabia has grave quality problems. To rectify this situation, it is essential to apply quality systems as tools for development. The SERVQUAL is one of these system options. It is used to gauge the service quality using five dimensions that have been time-tested since 1982. Thus, the significance of SERVQUAL in car care industry in Saudi Arabia cannot be overemphasized. The study further suggests that the SERVRQUAL dimension increases the perceived service quality, which in turn increases customer satisfaction. Thus, we proposed the following hypothesis:

**Hypothesis 2:** The perceived service quality of car care customers is positively linked with their satisfaction.

## Methods and Procedures

In this study, we employed a cross-sectional research design. Using a paper-pencil survey, data were collected form auto care workshops situated in the Eastern Province of Saudi Arabia. According to the study by Newsted et al. (1998), the survey method is valuable for assessing opinions and trends by collecting quantitative data. We adapted survey instruments from previous studies. The final survey was presented to a focus group of two Ph.D. marketing scholars who specialized in survey design marketing research. The survey was modified keeping in view the recommendations suggested by focus group members. We contacted the customers who used social media to check the updates and book the appointment for their vehicle’s service and maintenance. We abstained 130 surveys, 13 of which were excluded due to missing information. Therefore, the final sample encompassed 117 (26 female and 91 male) participants across multiple age groups: 10 aged less than 25 years, 46 aged between 26 and 30 years, 28 aged between 31 and 35 years, 21 aged between 36 and 40 years, and 12 aged older than 40 years (for details, refer to Table 1). Similarly, the averaged participants were graduates with more than 3 years of auto care service experience.

**TABLE 1:** Demographic information.

Characteristics	Number of respondents (*n* = 117)	
Gender	Male	26
	Female	91
Age	≤25.00	10
	26.00–30.00	46
	31.00–35.00	28
	36.00–40.00	21
	≥40.00	12
Qualification	Under graduation	30
	Graduation	63
	Above graduation	24
Experience of service	1–3	37
	4–6	57
	7–9	15
	≥10	08

### Measures

We measured service quality dimensions using 20 indicators. Customer satisfaction of the restaurant customers was assessed using 4-item scale (for detail, refer to Table 2). In this research, the 5-point Likert scale from 1 = strongly disagree to 5 = strongly agree was used.

**TABLE 2:** Constructs and items included in the questionnaire.

Construct	Dimension	Item	Measurement	References
Service quality	Tangibles of the auto care	TAC1	Workshop is convenient to customers	Parasuraman et al., 1988
		TAC2	Workshop is clean and tidy	
		TAC3	Workshop has good lighting and air circulation	
		TAC4	Workshop reception is easy to deal with	
	Reliability of the auto care	RAC1	Workshop has quick and accurate problem identification	Parasuraman et al., 1988
		RAC2	It is easy to communicate with workshop supervisors	
		RAC3	Workshop keeps and provides accurate cars records	
		RAC4	Workshop completes tasks as promised	
		RAC5	Workshop completes tasks correctly the first time	
		RAC6	Workshop shows concern for customers’ cars problems	
	Responsiveness of the auto care	REAC1	Workshop provides quick and accurate service	Parasuraman et al., 1988
		REAC2	Workshop solves customers’ complaints efficiently	
	Assurance of the auto care	AAC1	Workshop staff is knowledgeable	Parasuraman et al., 1988
		AAC2	I feel taken care of in this workshop	
	Empathy of the auto care	CAC1	The workshop cares about their customers	Parasuraman et al., 1988
		CAC2	It is easy to file a complaint in this workshop	
		CAC3	Workshop treats customers equally	
		CAC4	Workshop provides individualize attention to each customer	
		CAC5	Workshop treats customers with respect	
		CAC6	Workshop has customers’ best interest at heart	
	Customer satisfaction	CS1	How do you rate the physical appearance of the workshop?	Fornell et al., 1996
		CS2	How do you rate the care of reception in the workshop?	
		CS3	How do you rate your confidence in the workshop’s ability to deliver high quality services?	

### Control Variables

Following the previous research, customer’s gender and age were controlled to examine the influence of service quality dimensions on customer satisfaction.

## Data Analysis and Results

For data analysis and hypotheses testing, we employed the structural equation modeling (SEM) based on the partial least squares (PLS) in Smart-PLS. Smart-PLS 3 is a powerful tool, which is used for the confirmatory factor analysis (CFA) and SEM (Nachtigall et al., 2003). Research suggests that CFA is the best approach to examine the reliability and validity of the constructs. We employed SEM for hypotheses testing because it is a multivariate data analysis technique, which is commonly used in the social sciences (González et al., 2008).

### Common Method Bias

To ensure that common method bias (CMB) is not a serious concern for our results, we employed procedural and statistical and procedural remedies. During data collection, each survey in the research contained a covering letter explaining the purpose of the study and guaranteed the full anonymity of the participants. Moreover, it was mentioned in the cover letter that there was no right and wrong questions, and respondents’ answers would neither be related to their personalities nor disclosed to anyone. According to Podsakoff et al. (2003), the confidentiality of the responses can assist to minimize the possibility of CMB. Furthermore, CMB was verified through the Harman’s single-factor test (Podsakoff et al., 2003). All items in this research framework were categorized into six factors, among which the first factor explained 19.01% of the variance. Thus, our results showed that CMB was not an issue in our research. Moreover, using both tolerance value and the variance inflation factors (VIFs), we assessed the level of multicollinearity among the independent variables. Our results indicate that the tolerance values for all dimensions of service quality were above the recommended threshold point of 0.10 (Cohen et al., 2003), and VIF scores were between 1.4 and 1.8, which suggested the absence of multicollinearity; thus, it is not a serious issue for this study.

### Measurement Model

We performed CFA to analyze the reliability and validity of the constructs. The measurement model was assessed by examining the content, convergent, and discriminant validities. To assess the content validity, we reviewed the relevant literature and pilot test the survey. We used item loadings, Cronbach’s alpha, composite reliability (CR), and the average variance extracted (AVE) (Fornell and Larcker, 1981b) to assess the convergent validity. The findings of CFA illustrate that all item loadings are greater than 0.70. The acceptable threshold levels for all values were met, as the value of Cronbach’s alpha and CR was greater than 0.70 for all constructs (Fornell and Larcker, 1981b), and the AVE for all variables was above 0.50 (Tabachnick and Fidell, 2007; see Table 3). Thus, these findings show acceptable convergent validity.

**TABLE 3:** Item loadings, Cronbach’s alpha, composite reliability, and average variance extracted.

Variables	Item	Facto loadings	Cronbach’s alpha	CR	AVE
Tangibles of the auto care	TAC1	0.921		0.94	0.87
	TAC2	0.954			
	TAC3	0.922			
	TAC4	0.927			
Reliability of the auto care	RAC1	0.938		0.97	0.86
	RAC2	0.924			
	RAC3	0.818			
	RAC4	0.952			
	RAC5	0.932			
	RAC6	0.921			
Responsiveness of the auto care	REAC1	0.956		0.96	0.92
	REAC2	0.959			
Assurance of the auto care	AAC1	0.954		0.92	0.84
	AAC2	0.959			
Empathy of the auto care	CAC1	0.951		0.97	0.86
	CAC2	0.883			
	CAC3	0.934			
	CAC4	0.927			
	CAC5	0.926			
	CAC6	0.934			
Customer satisfaction	CS1	0.941		0.98	0.93
	CS2	0.966			
	CS3	0.971			
	CS4	0.975			

To analyze the discriminant validity, we evaluated the discriminant validity by matching the association between correlation among variables and the square root of the AVE of the variables (Fornell and Larcker, 1981a). The results demonstrate that the square roots of AVE are above the correlation among constructs, hence showing a satisfactory discriminant validity, therefore, indicating an acceptable discriminant validity. Moreover, descriptive statistics and correlations are provided in Table 4.

**TABLE 4:** Descriptive statistics and correlations.

Variables	*M*	SD	1	2	3	4	5	6	7	8
Gender	1.22	0.41	–							
Age	2.42	0.91	−0.101	–						
Education	0.84	0.37	0.124	0.101	–					
Tangibles of the auto care	3.97	0.98	0.097	–0.114	0.154*	–				
Reliability of the auto care	4.00	0.96	0.083	0.216	−0.091	0.611**	–			
Responsiveness of the auto care	3.94	1.06	0.123	–0.139	0.094	0.545**	0.631**	–		
Assurance of the auto care	4.03	1.01	0.127	0.154	0.063	0.676**	0.504**	0.518**	–	
Empathy of the auto care	3.98	0.98	0.103	0.177	0.194	0.484**	0.523**	0.611**	0.568**	–
Customer satisfaction	3.98	0.96	0.111	–0.106	0.081	0.345**	0.471**	0.567**	0.459**	0.464**

n = 117, *p < 0.05, **p < 0.01.

### Structural Model and Hypotheses Testing

**FIGURE 1:**
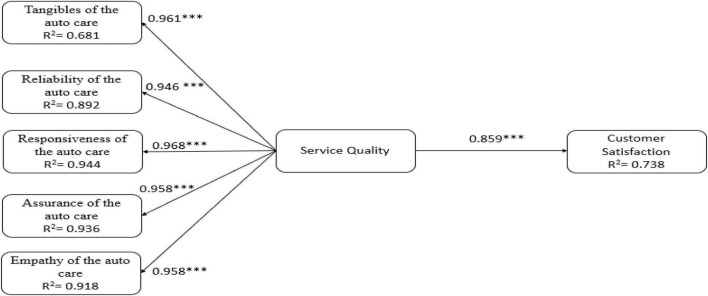
Results of the research model tests. ^***^p < 0.001.

After establishing the acceptable reliability and validity in the measurement model, we examined the relationship among variables and analyzed the hypotheses based on the examination of standardized paths. The path significance of proposed relations were calculated using the SEM through the bootstrap resampling technique (Henseler et al., 2009), with 2,000 iterations of resampling. The proposed research framework contains five dimensions of service quality (i.e., tangibles of the auto care, reliability of the auto care, responsiveness of the auto care, assurance of the auto care, and empathy of the auto care) and customer satisfaction of auto care. The results show that five dimensions of service quality are significantly related to customer’s perception of service quality of auto care; thus, hypotheses 1a, 1b, 1c, 1d, and 1e were supported. Figure 1 shows that the service quality of auto care is a significant determinant of customer satisfaction of auto care industry (β = 0.85, *p* < 0.001), supporting hypothesis 2. The result in Figure 1 also shows that 73.8% of the variation exists in customer satisfaction of auto care.

## Discussion

The main purpose of this research was to assess the relationship between service quality and customer satisfaction in the post pandemic world in Saudi Arabia. This study was designed to examine how satisfaction of auto care customers is influenced by service quality, especially, when pandemic was declared, and due to health concerns, the customers were reluctant to visit workshops physically (Mason et al., 2021). It appears that after the pandemic, customers were increasingly using online platforms for purchasing goods and services. This study reveals how customers of auto repair in Saudi perceive service quality and see how applicable SERVQUAL model across with five dimensions, including tangibles, responsiveness, reliability, assurance, and empathy measure service quality. The findings of this research show that five dimensions of SERVQUAL are positively related to the service quality perception of auto care customers in Saudi Arabia. Moreover, service quality perceptions are positively linked with customer satisfaction. These results indicate that auto care customers view service quality as an important antecedent of their satisfaction. The findings indicate that the customers perceive the service quality as a basic service expectation and will not bear the extra cost for this criterion. In this research, the positive connection between service quality and customer satisfaction is also consistent with previous studies (e.g., González et al., 2007; Gallarza-Granizo et al., 2020; Cai et al., 2021). Thus, service quality plays a key role in satisfying customers. These findings suggest that service organizations, like auto repair industry in Saudi Arabia could enhance satisfaction of their customers through improving service quality. Because of pandemic, people are reluctant to visit auto care workshops, and they try to book appointment through social media; so, by improving the quality of management of their social media pages, the workshops can provide accurate information for monitoring, maintaining, and improving service quality (Sofyani et al., 2020). More specifically, social media, which allows individuals to interact remotely, appears to be gaining significant importance as a tool for identifying customers’ products and service needs. Increasingly, customers are also increasingly engaging with retailers through social media to search and shop for product and services options, evaluate the alternatives, and make purchases.

Furthermore, the research on the customer service quality can be held essential since it acts as a means for the promotion of the competitiveness of an organization. Precisely, the knowledge about the customers’ view concerning service quality can be used by organizations as a tool to improve their customer services. For example, knowledge of the required customer service would help in the facilitation of training programs oriented toward the enlightenment of the overall employees on the practices to improve and offer high-quality customer services. Besides, information concerning customer services would be essential in decision-making process concerning the marketing campaigns of the firm, hence generating competitive advantage of the organization in the marketplace. Findings show that customers demand more from auto repair, so the company must work hard to increase all service quality dimensions to improve customer satisfaction. Thus, organizations ought to venture in customer services initiatives to harness high-quality services.

### Managerial Implications

The findings of this research indicate a strong association between SERVQUAL dimensions and perceived service quality. Perception of higher service quality leads to higher level of customer satisfaction among Saudi car care customers. In particular, the results indicate high scores for reliability, empathy, tangibles, and responsiveness. These are clear indications that the immense budgetary allocation has enabled these institutions to develop capacity. Nevertheless, the lack of a strong human resource base remains a key challenge in the car care industry. The effective use of social media plays a critical role in the responsiveness dimension of service quality. Companies need to develop their digital and social media marketing strategies in the post pandemic world to better satisfy their customers.

Saudi Arabia requires a large and well-trained human resource base. This requires intensive investment in training and development. Most of these workers have a limited contract, which reduced their focus on long-term dedication. Consequently, the government should provide longer-term contracts for workers in this critical sector. The contracts should include training on tailored courses to serve the identified needs in effective communication with the customers using digital media. We suggested that the auto car care workshops should provide training to their workers, particularly, on service technicians to enhance their skills that will help to deliver fast and reliable service to their auto customers.

Moreover, the auto car care workshops also provide customer care- or customer handling-related training especially for the service marketing personnel who handles customer directly for them to better understand the customer needs and expectations. This can be done at least once a year. This will help auto care workshops to improve their service quality.

## Limitation and Future Research Direction

This research is not without limitations. First, the findings of this study are based on data collected from a single source and at a single point of time, which might be subjected to CMB (Podsakoff et al., 2003). Future research can collect data from different points of time to validate the findings of this research. Second, this research was carried out with data obtained from Saudi auto car care customers; the findings of this research might be different because the research framework was retested in a different cultural context. Therefore, more research is needed to improve the understanding of the principles of service quality and customer satisfaction, as well as how they are evaluated, since these concepts are critical for service organizations’ sustainability and development. A greater sample size should be used in a similar study so that the findings could be applied to a larger population. Research on the effect of inadequate customer service on customer satisfaction, the impact of customer retention strategies on customer satisfaction levels, and the impact of regulatory policies on customer satisfaction is also recommended. Third, because most of the participants participated in this research are men, future studies should obtain data from female participants and provide more insights into the difference between male and female customers’ satisfaction levels. Moreover, due to limitation of time, the sample was collected from the eastern province. Consequently, further research should include a larger and more representative sample of the Saudi population. Because of the non-probability sampling approach used in this research, the results obtained cannot be generalized to a wide range of similar auto repair services situations, even though the methodology used in this study could be extended to these similar situations. Since the sample size considered is not that large, expectations could vary significantly. When compared with the significance of conducting this form of analysis, the limitations mentioned above are minor. Such research should be conducted on a regular basis to track service quality and customer satisfaction levels and, as a result, make appropriate changes to correct any vulnerability that may exist.

## Data Availability Statement

The original contributions presented in the study are included in the article/supplementary material, further inquiries can be directed to the corresponding author.

## Ethics Statement

Ethical review and approval was not required for the study on human participants in accordance with the local legislation and institutional requirements. Written informed consent for participation was not required for this study in accordance with the national legislation and the institutional requirements. The patients/participants provided their written informed consent to participate in this study.

## Author Contributions

SZ helped in designing the study. ZH helped in designing and writing the manuscript. MAA helped in data collection and analysis and writing the manuscript. SUR repositioned and fine-tuned the manuscript, wrote the introduction, and provided feedback on the manuscript.

## Conflict of Interest

The authors declare that the research was conducted in the absence of any commercial or financial relationships that could be construed as a potential conflict of interest.

## Publisher’s Note

All claims expressed in this article are solely those of the authors and do not necessarily represent those of their affiliated organizations, or those of the publisher, the editors and the reviewers. Any product that may be evaluated in this article, or claim that may be made by its manufacturer, is not guaranteed or endorsed by the publisher.
